# Atrial Fibrillation Detection from At-Rest PPG Signals Using an SDOF-TF Method

**DOI:** 10.3390/s26020416

**Published:** 2026-01-08

**Authors:** Mamun Hasan, Zhili Hao

**Affiliations:** Department of Mechanical and Aerospace Engineering, Old Dominion University, Norfolk, VA 23529, USA; mhasa004@odu.edu

**Keywords:** PPG signal, atrial fibrillation (AF), single-degree-of-freedom (SDOF) system, motion artifacts (MA), instant parameters, time-varying system parameters (TVSP), heart rate (HR), heart rate variability (HRV), respiration rate (RR), respiration modulation (RM)

## Abstract

At-rest PPG signals have been explored for detecting atrial fibrillation (AF), yet current signal-processing techniques do not achieve perfect accuracy even under low-motion artifact (MA) conditions. This study evaluates the effectiveness of a single-degree-of-freedom time–frequency (SDOF-TF) method in analyzing at-rest PPG signals for AF detection. The method leverages the influence of MA on the instant parameters of each harmonic, which is identified using an SDOF model in which the tissue–contact–sensor (TCS) stack is treated as an SDOF system. In this model, MA induces baseline drift and time-varying system parameters. The SDOF-TF method enables the quantification and removal of MA and noise, allowing for the accurate extraction of the arterial pulse waveform, heart rate (HR), heart rate variability (HRV), respiration rate (RR), and respiration modulation (RM). Using data from the MIMIC PERform AF dataset, the method achieved 100% accuracy in distinguishing AF from non-AF cases based on three features: (1) RM, (2) HRV derived from instant frequency and instant initial phase, and (3) standard deviation of HR across harmonics. Compared with non-AF, the RM for each harmonic was increased by AF. RM exhibited an increasing trend with harmonic order in non-AF subjects, whereas this trend was diminished in AF subjects.

## 1. Introduction

Atrial fibrillation (AF) is a common cardiac arrhythmia that poses significant health risks, including stroke and heart failure, making timely and accurate detection crucial for mitigating these risks [1,2,3,4,5]. Compared to the gold-standard electrocardiogram (ECG) for AF detection, photoplethysmography (PPG) has emerged as a viable alternative that allows for the routine monitoring of cardiovascular (CV) health in clinical settings or at home due to its accessibility and ease of use [1,2,3,4,5]. When utilizing PPG sensors for AF detection, measurements can be categorized into two distinct types: wearable PPG sensors at the wrist during movement [1,2] and non-wearable PPG sensors on the finger at rest [6,7,8,9,10,11,12,13]. The shift towards wearable PPG signals for AF detection is driven by the practical benefits of continuous monitoring [1,2]; however, these signals typically exhibit lower fidelity and are more susceptible to high motion artifacts (MAs), resulting in significantly lower detection accuracy compared to at-rest PPG signals [1,2,14,15].

While at-rest PPG signals are much less affected by MAs than their wearable counterparts, they still do not achieve a perfect match with the gold-standard ECG, primarily due to three factors: MA, noise, and individual variability (i.e., physiological condition) [3,4,5,16,17,18]. Improving the precision of at-rest PPG analysis is essential for enabling cost-effective and scalable AF screening in both clinical and home environments. Thus, there is a critical need to develop more effective signal-processing algorithms to achieve a 100% accuracy rate for at-rest PPG signals, reducing false positives and negatives to ensure timely and accurate detection and treatment [2,9,19,20].

Among the datasets for at-rest PPG signals used in AF detection, the MIMIC PERform AF dataset, available on the PhysioNet website [21], has been extensively studied for testing various signal-processing techniques [6,7,8,9,10,11,12,13]. Despite the relatively high quality (i.e., low MA) of these at-rest signals, no signal-processing techniques have yet achieved perfect accuracy for AF detection [6,7,8,9,10,11,12]. Traditional time-domain techniques have been employed to identify irregularities in the PPG signals [6,7]. In addition, time–frequency techniques have been utilized to decompose the PPG signals into different frequency components, allowing for improved noise reduction and feature extraction [9]. Recently, machine learning algorithms have gained prominence due to their ability to learn complex patterns from data [11,12]. Deep learning techniques have also been implemented, which capitalize on their capability to automatically extract relevant features from PPG signals, with reported accuracy rates reaching 98% for this dataset [8]. However, deep learning techniques often function like a black box, lacking interpretability and transparency, which can make it challenging for clinicians to trust their results [22,23,24]. Moreover, these techniques require training on specific datasets, raising concerns about the generalizability of findings across diverse demographic groups, highlighting the need for proper validation to ensure their widespread clinical applicability [22,23,24]. Above all, the persistent, albeit small, margin of error underscores the necessity for further improvement in signal-processing techniques on at-rest PPG signals to attain clinical-grade accuracy [19,20].

This study aims to apply a generalized single-degree-of-freedom (SDOF) time–frequency (SDOF-TF) method [25] to the at-rest PPG signals in the MIMIC PERform AF dataset to examine the effectiveness of this method in removing MA and noise from these pulse signals for AF detection. The SDOF-TF method is built on an SDOF model of MA in a measured pulse signal, where the tissue–contact–sensor (TCS) stack, sandwiched between an artery and the PPG sensor at the skin’s surface, is treated as an SDOF system [26]. MA causes a baseline drift (BD), which refers to the time-varying distance change in the PPG sensor relative to the artery, and, consequently, time-varying system parameters (TVSPs) of the TCS stack [27,28]. While BD manifests as additive noise, which is low-frequency (<0.7 Hz) and can be easily removed, TVSP-generated distortion manifests as multiplicative noise that rides on each harmonic in a PPG signal [25,27,28]. In [25], the working principle of the SDOF-TF method was detailed and applied to analyze three pulse signals measured using a tactile sensor and four PPG signals under varying physiological conditions (note: of the four PPG signals, one is from the AF group and one is from the non-AF group within the MIMIC PERform AF dataset); the effectiveness and generalizability of the method in removing MA and noise from these signals measured using both sensor types were qualitatively demonstrated by the consistency of the derived differences between physiological conditions with the related findings in the literature. In particular, several extracted parameters in [25] revealed significant differences between the AF and the non-AF subjects, but these differences lacked statistical significance.

As compared to the previous study [25], which focused on the SDOF-TF method itself—its working principle, generalizability to different sensor types, and qualitative validation—the original contributions of this study include (1) applying the SDOF-TF method to the entire MIMIC PERform AF dataset, enabling the quantitative validation of its effectiveness in removing MA and noise from at-rest PPG signals; (2) identifying all extracted parameters that show non-overlapping values between the AF and non-AF groups, thereby serving as statistically significant indices for AF detection; and (3) revealing the physiological implications of some of the observed differences between AF and non-AF subjects.

## 2. Materials and Methods

### 2.1. An SDOF-TF Method for Time-Frequency Analysis

#### 2.1.1. An SDOF Model of MA in a PPG Signal

**Figure 1:**
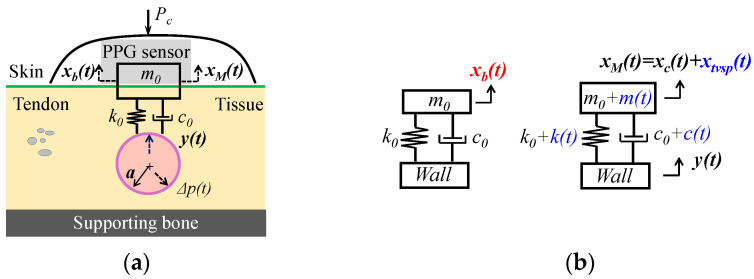
The tissue–contact–sensor (TCS) stack between a PPG sensor and an artery. (**a**) Schematic; (**b**) an SDOF model of MA in a PPG signal.

The SDOF model of MA in a PPG signal is based on three assumptions: (1) the tissue–contact–sensor (TCS) stack behaves linearly during pulse signal measurement and (2) the effect of the TCS stack in the true pulse signal is negligible, and thus the input pulse signal to the TCS stack is the arterial wall displacement *y*(*t*) instead of the pulsatile pressure *Δp*(*t*), as shown in Figure 1a.

A PPG sensor contains a light emitter and a photodetector. As shown in Figure 1a, a tape (or a mechanical fixture) is used to fix a PPG sensor with contact pressure *P_c_* for establishing the tissue–sensor contact. Due to the deformability of the tissue above the artery, the tissue–contact–sensor (TCS) stack is treated as an SDOF system with spring stiffness *k*_0_, damping coefficient *c*_0_, and mass *m*_0_, to capture its dynamic behavior during pulse measurement. The PPG sensor forms part of *m*_0_. Note that *P_c_* presets the nominal values of *m*_0_, *k*_0_, and *c*_0_ and is fully accounted for by the three system parameters. The light sent by the light emitter passes through the TCS stack and is partially absorbed by blood in the artery, and the transmitted or reflected light is detected by the photodetector as the measured pulse signal (i.e., PPG signal). Optical transduction in a PPG signal is extremely complex [4]. For simplicity, optical transduction is neglected here, except when considering the effect of MA on a PPG signal, and thus a PPG signal is represented as the displacement at the mass.

Figure 1b shows the SDOF model of MA in a PPG signal. Due to MA, the PPG sensor itself encounters a time-varying displacement *x_b_*(*t*) (i.e., BD) at the mass, which further induces time-varying system parameters (TVSPs): *m*(*t*), *k*(*t*), and *c*(*t*) of the TCS stack:

(1) $$m=m_{0}+m(t),\;c=c_{0}+c(t),\;k=k_{0}+k\left(t\right)\;\mathrm{with}\;m\left(t\right),\;k(t),\;c(t)\propto x_{b}(t)$$

The true pulse signal y(t) in an artery serves as the base excitation for the SDOF system and is time-harmonic [28,29]:

(2) $$y\left(t\right)=y_{0}e^{j{(\omega }_{y}t+\varphi_{y})}$$

where $y_{0}$, $\varphi_{y}$, and $\omega_{y}$ are the amplitude, phase, and angular frequency of y(t), respectively. y(t) causes displacement $x_{M}(t)$ at the mass [25,27,28]:

(3) $$\left[m_{0}+m\left(t\right)\right]\cdot \frac{d^{2}x_{M}\left(t\right)}{{dt}^{2}}+\left[c_{0}+c\left(t\right)\right]\cdot \frac{dx_{M}\left(t\right)}{dt}+\left[k_{0}+k\left(t\right)\right]\cdot x_{M}\left(t\right)=\left[k_{0}+k\left(t\right)\right]\cdot y\left(t\right)+\left[c_{0}+c(t)\right]\cdot \frac{dy\left(t\right)}{dt}$$

Due to TVSP in Equation (3), $x_{M}\left(t\right)$ is non-stationary and takes the following form [25]:

(4) $${x_{M}(t)=x}_{T}(t)e^{j\varphi_{T}\left(t\right)}\;\;\mathrm{with}\;\omega_{T}(t)=\frac{{d\varphi }_{T}(t)}{dt}$$

where $x_{T}$, $\varphi_{T}$, and $\omega_{T}$ are the instant amplitude, phase, and frequency of $x_{M}(t)$, respectively. The PPG signal $x_{PPG}\left(t\right)\;$ becomes [25]

(5) $$x_{PPG}\left(t\right)=x_{M}\left(t\right)+x_{b}(t)\;\mathrm{with}\;x_{M}\left(t\right)=x_{C}\left(t\right)+x_{tvsp}(t)$$

where $x_{b}(t)$ is BD; $x_{C}\left(t\right)$ is the measured pulse signal when free of MA (i.e., free of TVSP); and $x_{tvsp}(t)$ is the TVSP-generated distortion in Equation (3). When free of MA, based on Equation (3), the measured pulse signal $x_{C}\left(t\right)$ is

(6) $$x_{C}\left(t\right)=x_{0}e^{j{(\omega }_{x}t+\varphi_{x})}=G_{0}e^{j\varphi_{0}}y_{0}e^{j{(\omega }_{y}t+\varphi_{y})}$$

(7) $$\;\mathrm{with}\;G_{0}e^{j\varphi_{0}}\;=\frac{k_{0}+c_{0}j\omega_{y}}{{-m}_{0}\omega_{y}^{2}+c_{0}j\omega_{y}+k_{0}}\omega_{x}=\omega_{y};\;\varphi_{x}=\varphi_{y}+\varphi_{0};\;x_{0}=G_{0}y_{0}$$

Thus, the total distortion caused by MA in a PPG signal is

(8) $$x_{PPG-MA}\left(t\right)=x_{tvsp}\left(t\right)+x_{b}(t)\;\mathrm{with}\;x_{TVSP}\left(t\right)=x_{M}\left(t\right)-x_{C}\left(t\right)$$

While *x_b_*(*t*) is a low-frequency additive noise (<0.7 Hz), *x_tvsp_*(*t*) rides on each harmonic of the true pulse signal, as shown in Equation (3), and thus is multiplicative noise.

The further analysis of *x_tvsp_*(*t*) [25] reveals that *x_tvsp_*(*t*) (1) dramatically swings the instant amplitude, (2) slightly affects the instant frequency, and (3) almost has no effect on the instant initial phase of each harmonic in *x_M_*(*t*). Based on these identified effects, an SDOF-TF method is developed for the removal of MA (i.e., *x_b_*(*t*) + *x_tvsp_*(*t*)) and noise from a PPG signal and the extraction of arterial pulse waveform (APW), heart rate (HR), and respiration parameters.

#### 2.1.2. An SDOF-TF Method

**Figure 2:**
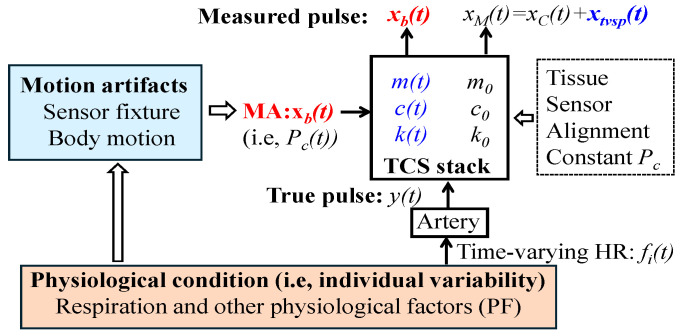
All the factors (except sensor noise) involved in PPG signal measurement and their quantitative manifestation in a PPG signal via the SDOF model of MA in a PPG signal.

As shown in Figure 2, the tissue, sensor, alignment, and constant *P_c_* (i.e., sensor fixture) involved during pulse measurement cannot be independently quantified. Instead, their collective behavior can be quantified as the nominal parameters *m*_0_, *c*_0_, and *k*_0_ of the SDOF system representing the TCS stack. The physiological condition of an individual (i.e., individual variability) manifests in a PPG signal in two ways. First, physiological condition governs time-varying HR, and thus manifests as time-varying frequency *f_i_*(*t*) of the *i*th harmonic in the true pulse signal *y*(*t*). Second, it influences body motion, which, in conjunction with the sensor fixture, gives rise to MA. MA induces BD *x_b_*(*t*)—equivalent to a time-varying *P_c_*(*t*)—and TVSP-generated distortion *x_tvsp_*(*t*). Consequently, *x_b_*(*t*) and *x_tvsp_*(*t*) collectively quantify the inseparable effect of the physiological condition and sensor fixture on a PPG signal, from the perspective of MA formation in a PPG signal.

From the perspective of the time-varying HR of an arterial pulse signal, physiological condition includes respiration and other physiological factors (PFs) [30]. While the effect of respiration on time-varying HR is commonly assumed to be time-harmonic, the effect of PF is deemed to be non-time-harmonic. The true pulse signal *y*(*t*) is a collection of multiple harmonics of HR [30]:

(9) $$y\left(t\right)=\sum_{i=1}^{N}A_{i}\cdot cos(2\pi {if}_{0}t+\phi_{0i}+\int \psi \left(t\right))\;\mathrm{with}\;\psi \left(t\right)=B/f_{r}\cdot cos(2\pi f_{r}t+\alpha_{0})$$

where *f*_0_ is the frequency of constant HR, *A_i_* and *ϕ*_0i_ are the amplitude and initial phase of the *i*th harmonic, respectively, and $\int \psi \left(t\right)$ is related to respiration. Note that *A_i_*, *f*_0_, and *ϕ*_0i_ are all constant, and the effect of PF on HR is excluded in Equation (9). The frequency of the *i*th harmonic is altered by respiration as *i·f*_0_ + *B·*cos (2π*f_r_t + α*_0_), where *B* is respiration modulation (RM), indicating the strength of respiratory sinus arrhythmia (RSA) on altering HR [30], and *f_r_* and *α*_0_ are the respiration rate (RR) and initial phase of respiration, respectively.

**Figure 3:**
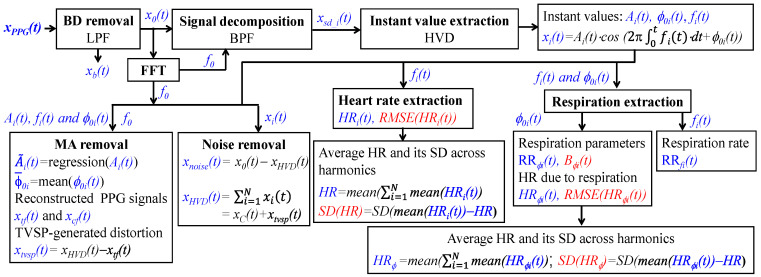
A block diagram of the SDOF-TF method for quantifying and removing MA and noise from a PPG signal and extraction of APW, HR, and respiration parameters (note that the parameters in red font are the identified indices for AF detection with 100% accuracy).

Figure 3 illustrates a simplified signal-processing algorithm of the SDOF-TF method. The detailed algorithm can be found in [25]. A PPG signal *x_PPG_*(*t*) goes through a low-pass filter (LPF) to remove *x_b_*(*t*) and obtain *x*_0_(*t*):

(10) $$x_{0}\left(t\right)=x_{C}\left(t\right)+x_{TVSP}\left(t\right)+x_{noise}\left(t\right)$$

where *x_noise_*(*t*) denotes noise associated with the sensor.

Fast Fourier transform (FFT) is conducted on *x*_0_(*t*) to obtain the frequency of the first harmonic of the PPG signal. Then, a bandpass filter (BPF) is used to separate the *i*th harmonic *x_sd_i_*(*t*) from *x*_0_(*t*). Hilbert vibration decomposition (HVD) [28] is used to extract instant amplitude *A_i_*(*t*), instant frequency *f_i_*(*t*), and instant initial phase *ϕ*_0*i*_(*t*)of the *i*th harmonic, whose sum is denoted as *x_HVD_*(*t*) and is equal to *x_C_*(*t*) +*x_tvsp_*(*t*) in Equation (5):

(11) $$x_{HVD}\left(t\right)=\sum_{i=1}^{N}x_{i}\left(t\right)=x_{C}\left(t\right)+x_{tvsp}\left(t\right)\;\mathrm{with}\;x_{i}\left(t\right)=A_{i}\left(t\right)\cdot cos(2\pi \cdot \int f_{i}\left(t\right)dt+\phi_{0i}(t))$$

As compared to *x_sd_i_*(*t*), sensor noise is greatly alleviated in *x_i_*(*t*) [25,28]. The sensor noise is then calculated as

(12) $$x_{noise}\left(t\right)=x_{0}\left(t\right)-x_{HVD}\left(t\right)$$

To remove MA (i.e., *x_tvsp_*(*t*)), the regression line of *A_i_*(*t*): ${\tilde{A}}_{i}\left(t\right)$ and the mean of *ϕ*_0*i*_(*t*): ${\bar{\phi }}_{0i}$ are obtained. Then, free of MA and noise, the pulse signal *x_tf_*(*t*) with time-varying frequency (or HRV) is reconstructed [25]:

(13) $$x_{tf}\left(t\right)=\sum_{i=1}^{N}x_{tf\_i}\left(t\right)\;with\;x_{tf\_i}\left(t\right)={\tilde{A}}_{i}\left(t\right)\cdot cos(2\pi \cdot \int f_{i}\left(t\right)dt+{\bar{\phi }}_{0i})\;$$

To examine the effect of time-varying frequency (or HRV) on the APW of a PPG signal, the corresponding pulse signal *x_cf_*(*t*) with constant frequency (i.e., constant HR) is reconstructed [25]:

(14) $$x_{cf}\left(t\right)=\sum_{i=1}^{N}x_{cf\_i}\left(t\right)\;with\;x_{cf\_i}\left(t\right)={\tilde{A}}_{i}\left(t\right)\cdot cos(2\pi \cdot f_{0}+{\bar{\phi }}_{0i})$$

Accordingly, *x_tvsp_*(*t*) riding on each harmonic in a PPG signal is calculated as

(15) $$x_{tvsp}\left(t\right)=x_{HVD}\left(t\right)-x_{tf}\left(t\right)$$

We further extract *HR_i_*(*t*) from the instant frequency *f_i_*(*t*) of the *i*th harmonic [25]. Note that *HR_i_*(*t*) represents the total HR, which accounts for the effects of both respiration and PF on HR. HRV is calculated using root mean squared error (RMSE). The total HVR is then calculcated as *RMSE*(*HR_i_*(*t*)).

To calculate one value for the total HR of a subject from the three harmonics, the average HR of each harmonic is calculated as mean (*HR_i_*(*t*)). The HR of a subject is then calculated as the average of the averaged HR of the three harmonics, and the difference in average HR between the three harmonics is calculated as their standard deviation (SD):

(16) $$HR=mean(\sum_{i=1}^{N}{mean(HR}_{i}\left(t\right)))SD\left(HR\right)=SD[mean\left({HR}_{i}\left(t\right))-HR\right]\;$$

Extraction of the respiration signal is conducted on both *f_i_*(*t*) and *ϕ*_0*i*_(*t*) [25]. While the RR extracted from *f_i_*(*t*) is denoted as *RR_fi_*(*t*), the RR extracted form *ϕ*_0*i*_(*t*) is denoted as *RR_ϕi_*(*t*). As will be seen in Section 3, *RR_fi_*(*t*) underestimates the RR, as compared to *RR_ϕi_*(*t*). Thus, the RM is only extracted from *ϕ*_0*i*_(*t*) and denoted as *B_ϕi_*(*t*).

The HR accounting solely for the effect of respiration on HR is also calculated and is denoted as *HR_ϕi_*(*t*), and the associated HRV is calculated as *RMSE* (*HR_ϕi_*(*t*)). Similarly to *HR* and *SD* (*HR*) from *f_i_*(*t*) in Equation (16), we also calculate one value for the HR, which accounts solely for respiration, of a subject from the three harmonics:

(17) $${HR}_{\emptyset }=mean(\sum_{i=1}^{N}{mean(HR}_{\emptyset i}\left(t\right))\;SD({HR}_{\emptyset })=SD[mean\left({HR}_{\emptyset i}\left(t\right))-{HR}_{\emptyset }\right]$$

where *HR_ϕ_* denotes the average value from the three harmonics and *SD* (*HR_ϕ_*) is their standard deviation of the three harmonics.

### 2.2. PPG Signals of AF and Non-AF Subjects and Their Analysis

To evaluate the effectiveness of the SDOF-TF method in removing MA and noise from a PPG signal measured at rest, we choose to apply it to analyze the at-rest PPG signals in the MIMIC PERform AF Dataset [21], which contains data recorded from 35 critically ill adults during routine clinical care using a bedside monitor. Each subject has 20 min of data sampled at 125 Hz. The PPG signals of all subjects in the dataset are labeled as either AF or non-AF. However, the PPG signals for one subject in the non-AF group and two subjects in the AF group are missing (see Table A1 in Appendix A). Thus, there are 14 subjects in the non-AF group and 18 subjects in the AF group.

The signal-processing algorithm for implementing the SDOF-TF method was developed in MATLAB2025a and follows the procedure described in [25]. Compared with [25], four additional parameters—average HR and its SD across harmonics from the instant frequency and instant initial phases—are extracted from a PPG signal. An 80 s segment with no abrupt change was selected from each PPG signal for analysis (see Table A1 in Appendix A). Only the first three harmonics of the PPG signals were analyzed, since the higher harmonics in the PPG signals of the AF group were extremely small, compared with their lower counterparts. As will be seen in Section 3, multiple extracted parameters can effectively distinguish between the AF and non-AF groups with 100% accuracy; therefore, no further statistical analysis is necessary. All the figures illustrating the analyzed results were generated using the same software (see Table A2 and Table A3 in Appendix A for the numerical values corresponding to these figures).

## 3. Results

### 3.1. Examination of MA, Noise, APW, HR, and Respiration Parameters

As the detailed analysis of the first subject in each group has been previously reported in [25], the present work focuses on the intermediate results of the SDOF-TF method from the second subject in each group. This aims to elucidate the effects of MA and noise on the PPG signals under two physiological conditions: non-AF versus AF, and to further enhance the interpretability and transparency of the method.

**Figure 4:**
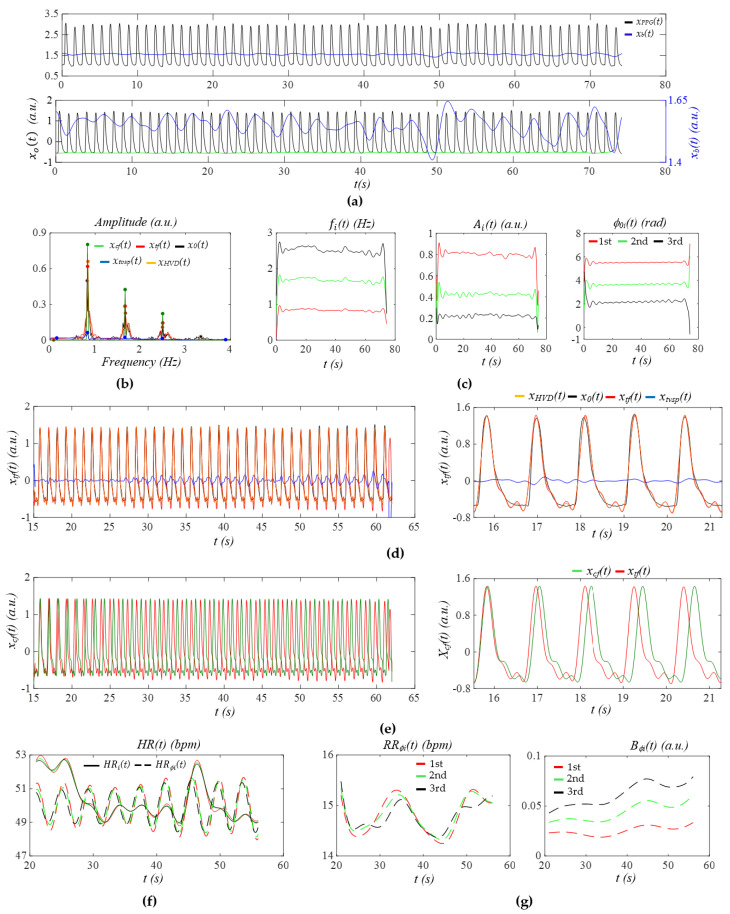
The PPG signal (segment: 945~1025 s) of Subject 2 in the non−AF group: (**a**) *x_PPG_*(*t*), *x*_0_(*t*), and *x_b_*(*t*) (green line: baseline of *x*_0_(*t*)); (**b**) frequency spectra of *x*_0_(*t*), *x_HVD_*(*t*)*, x_tf_*(*t*), *x_cf_*(*t*), and *x_tvsp_*(*t*); (**c**) instant parameters of the harmonics; (**d**) reconstructed pulse signal *x_tf_*(*t*) with *f_i_*(*t*); (**e**) reconstructed pulse signal *x_cf_*(*t*) with *f*_0_; (**f**) *HR_i_*(*t*) and *HR_ϕi_*(*t*); and (**g**) *ϕ*_0*i*_(*t*) −based respiration parameters.

Figure 4a shows the PPG signal (segment: 945~1025 s) of Subject 2 in the non-AF group. After the removal of *x_b_*(*t*), all the pulse cycles are aligned to a similar level. Except the pulse cycles around 50 s, which experience relatively large MA, MA in the remaining pulse cycles is minimal. Figure 4b presents the frequency spectrum of *x*_0_(*t*), *x_tf_*(*t*), *x_HVD_*(*t*), *x_cf_*(*t*)*,* and *x_tvsp_*(*t*), revealing small sidebands from HRV and a small *x_tvsp_*(*t*). In Figure 4c, the relatively large MA around 50 s causes noticeable changes in *f_i_*(*t*) and *A_i_*(*t*), but no change in *ϕ*_0*i*_(*t*). Compared to *f_i_*(*t*), *ϕ*_0*i*_(*t*) exhibits a more consistent time-harmonic behavior, capturing the respiration signal far more effectively.

Figure 4d compares the reconstructed pulse signal with time-varying frequency *x_tf_*(*t*) with *x_HVD_*(*t*), *x*_0_(*t*), and *x_tvsp_*(*t*). The difference between *x*_0_(*t*) and *x_HVD_*(*t*) represents noise, which obscures the dicrotic notch. The small difference between *x_tf_*(*t*) and *x_HVD_*(*t*) indicates small MA (or small *x_tvsp_*(*t*)). As shown in Figure 4e, due to low HRV, the difference in APW between *x_cf_*(*t*) and *x_tf_*(*t*) is very small, except that there is a large phase shift between them, possibly due to the large swing of *HR_i_*(*t*), as shown in Figure 4f. Note that in Figure 4f, *HR_i_*(*t*) and *HR_ϕi_*(*t*) from the three harmonics are almost identical. The difference between *HR_i_*(*t*) and *HR_ϕi_*(*t*) is believed to arise from the fact that *HR_i_*(*t*) reflects the combined effects of respiration and PF on HR, whereas *HR_ϕi_*(*t*) captures only the influence of respiration. Furthermore, *HR_i_*(*t*) is affected by MA to a larger extent than *HR_ϕi_*(*t*) [25]. Additionally, *HR_ϕi_*(*t*) exhibits a clear time-harmonic pattern. Figure 4g presents the instant RR *RR_ϕi_*(*t*) and instant RM *B_ϕi_*(*t*) extracted from *ϕ_i_*(*t*). While *RR_ϕi_*(*t*) does not vary with harmonic order, *B_ϕi_*(*t*) shows an increasing trend with harmonic order.

**Figure 5:**
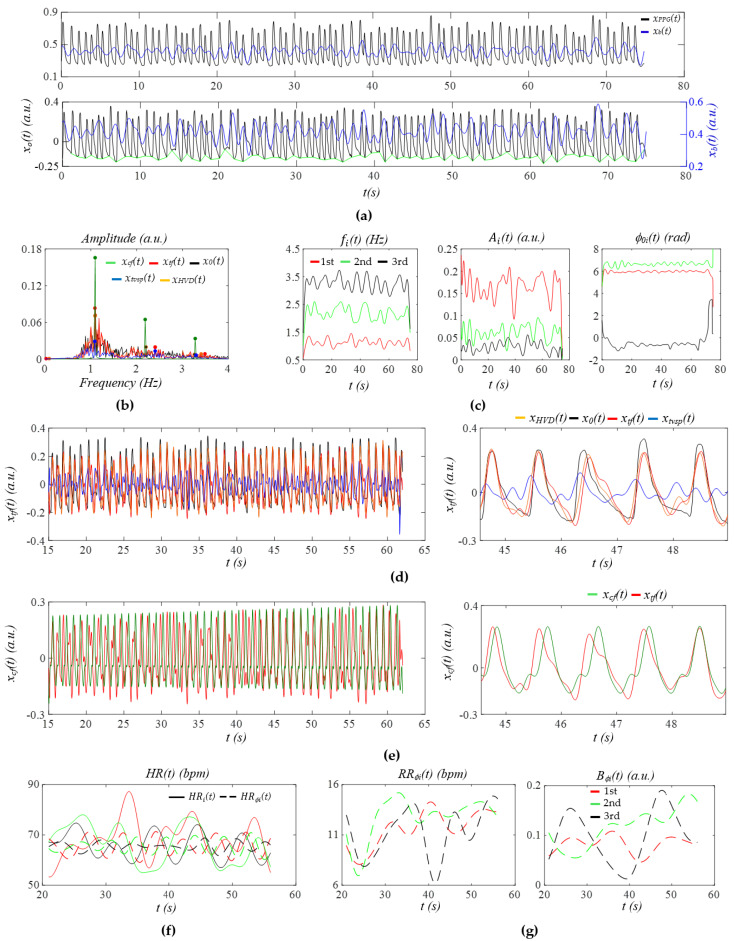
The PPG signal (segment: 955~1035 s) of Subject 2 in the AF group: (**a**) *x_PPG_*(*t*), *x*_0_(*t*), and *x_b_*(*t*) (green line: baseline of *x*_0_(*t*)); (**b**) frequency spectra of *x*_0_(*t*), *x_HVD_*(*t*)*, x_tf_*(*t*), *x_cf_*(*t*), and *x_tvsp_*(*t*); (**c**) instant parameters of the harmonics; (**d**) reconstructed pulse signal *x_tf_*(*t*) with *f_i_*(*t*); (**e**) reconstructed pulse signal *x_cf_*(*t*) with *f*_0_; (**f**) *HR_i_*(*t*) and *HR_ϕi_*(*t*); and (**g**) *ϕ*_0*i*_(*t*)−based respiration parameters.

Figure 5a shows the PPG signal (segment: 955~1035 s) from Subject 2 in the AF group. Compared to Figure 4a, *x_b_*(*t*) exhibits pronounced temporal variations, with an amplitude ranging from one-third to one-half of the pulse signal amplitude. As shown in Figure 5b, the large and wide sidebands of *x_tf_*(*t*) indicate substantial HRV, and the wide and relatively large sidebands of *x_tvsp_*(*t*) reflect large MA, compared to Figure 4b. Similarly to Figure 4c, *ϕ_i_*(*t*) in Figure 5c captures the respiration signal more effectively than *f_i_*(*t*).

As shown in Figure 5d, the moderate difference between *x_tf_*(*t*) and *x_HVD_*(*t*) indicates a relatively large *x_tvsp_*(*t*). As shown in Figure 5e, the substantial changes in the time-varying frequency (i.e., large HRV) result in pronounced variations in the pulse waveform between cycles. Figure 5f highlights that the remarkable difference between *x_cf_*(*t*) and *x_tf_*(*t*) underscores the significant role of HRV in preserving the dicrotic notch in the diastolic portion of the pulse waveform. As shown in Figure 5f, *HR_i_*(*t*) and *HR_ϕi_*(*t*) are comparable in amplitude and their time-varying patterns are remarkably misaligned across the harmonics. Figure 5g reveals that both *RR_ϕi_*(*t*) and *B_ϕi_*(*t*) do not exhibit any changing trends with harmonic order.

**Figure 6:**
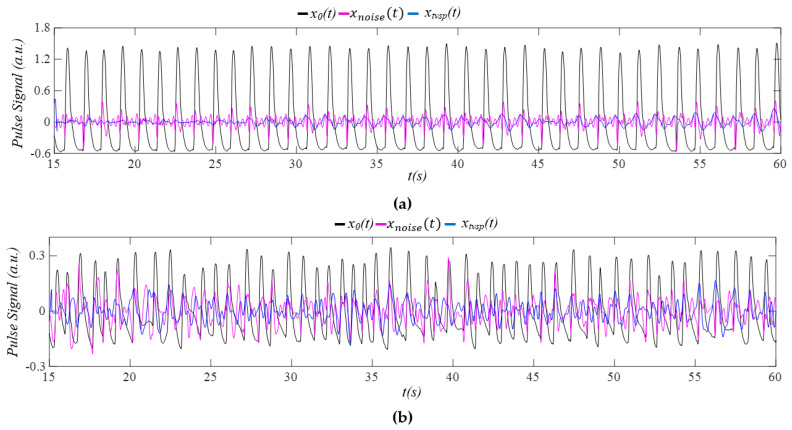
Comparison of *x_tvsp_*(*t*) versus *x_noise_*(*t*) in the PPG signal *x*_0_(*t*) free of BD *x_b_*(*t*): (**a**) Subject 2 in the non−AF group; (**b**) Subject 2 in the AF group.

Figure 6 compares noise *x_noise_*(*t*) and MA *x_tvsp_*(*t*) with *x*_0_(*t*). As shown in Figure 6a, *x_noise_*(*t*) exhibits a large spike at the peak of each pulse cycle in the non−AF subject. The magnitude of *x_noise_*(*t*) is comparable to that of *x_tvsp_*(*t*). Interestingly, both *x_noise_*(*t*) and *x_tvsp_*(*t*) display time−varying characteristics. In contrast to the AF subject shown in Figure 6b, *x_noise_*(*t*) and *x_tvsp_*(*t*) are similar in magnitude and both demonstrate random time−varying behavior. These two signals are relatively large, as compared to *x*_0_(*t*).

**Figure 7:**
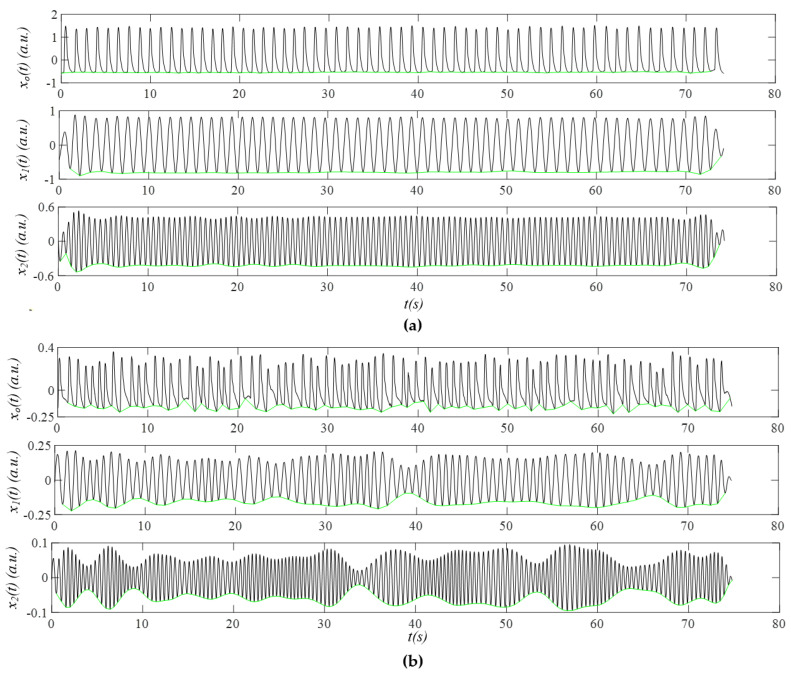
The signals of *x*_0_(*t*), the first harmonic *x*_1_(*t*), and the second harmonic *x*_2_(*t*): (**a**) Subject 2 in the non−AF group; (**b**) Subject 2 in the AF group (note: green line—the baseline of a signal connecting the start and end points of pule cycles).

**Figure 8:**
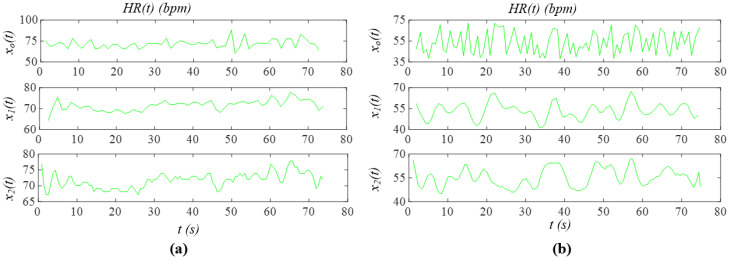
Extraction of HR in the time domain from *x*_0_(*t*), *x*_1_(*t*), and *x*_2_(*t*) in Figure 7: (**a**) Subject 2 in the non-AF group; (**b**) Subject 2 in the AF group.

Figure 7 shows *x*_0_(*t*), the first harmonic *x*_1_(*t*), and the second harmonic *x*_2_(*t*) (see Equation (11)). While *x*_0_(*t*) includes both *x_noise_*(*t*) and *x_tvsp_*(*t*), *x*_1_(*t*) and *x*_2_(*t*) contain only *x_tvsp_*(*t*). The baseline connecting the start and end points of the pulse cycles are used to extract HR from these cycles in the time domain. As shown in Figure 7a, due to small MA and low HRV in the non−AF subject, the baselines for *x*_0_(*t*) and *x*_1_(*t*) remain nearly flat. In contrast, the baseline for *x*_2_(*t*) exhibits discernable variation between pulse cycles, as the smaller amplitude of *x*_2_(*t*) makes it more sensitive to MA and HRV. As shown in Figure 7b, large MA and high HRV in the AF subject produce substantial baseline variation across pulse cycles for all three signals: *x*_0_(*t*), *x*_1_(*t*), and *x*_2_(*t*). Figure 8 shows the extracted HR based on the baselines in Figure 7. As compared to the non-AF subject, the HR extracted from *x*_0_(*t*) in the AF subject exhibits pronounced temporal variation. The HR time-varying pattern derived from both *x*_1_(*t*) and *x*_2_(*t*) varies less in the non-AF subject than in the AF subject.

**Figure 9:**
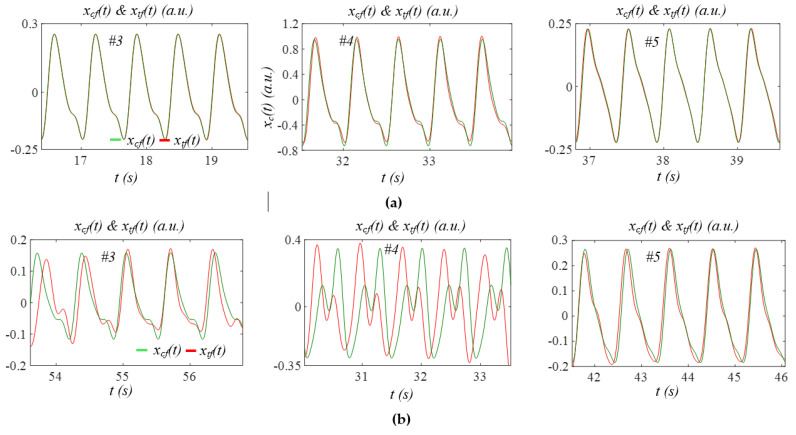
Comparison of APW between time-varying HR and constant HR: (a) *x_tf_*(*t*) and *x_cf_*(*t*) of Subjects 3, 4, and 5 in the non−AF group; (b) *x_tf_*(*t*) and *x_cf_*(*t*) of Subjects 3, 4, and 5 in the AF group.

Figure 9 shows additional extracted APWs from subjects in each group. For non-AF subjects, low HRV results in *x_tf_*(*t*) and *x_cf_*(*t*) are nearly identical. In AF subjects, high HRV leads to a pronounced difference between *x_cf_*(*t*) and *x_tf_*(*t*). The observed small difference between *x_tf_*(*t*) and *x_cf_*(*t*) in Subject 5 in the AF group arises from the fact that this subject exhibits the lowest HRV among all the AF subjects, as will be seen later. Finally, the APW varies dramatically between subjects in each group.

### 3.2. Comparison of AF Versus Non−AF Groups’ Extracted Parameters

**Figure 10:**
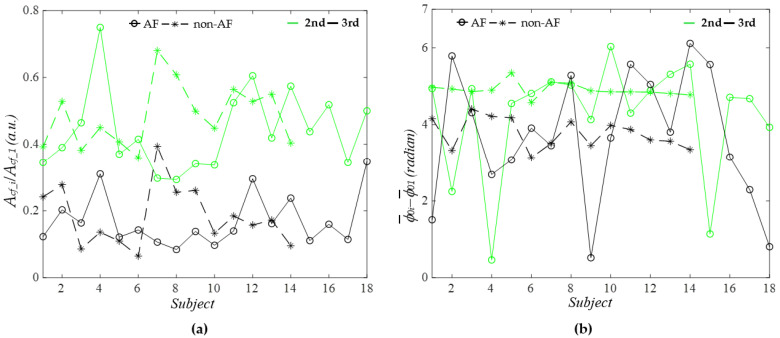
Comparison of AF versus non−AF *A_cf_i_*/*A_cf__*_1_ and $\bar{\phi }$_0*i*_ − $\bar{\phi }$_01_ of the harmonics of the PPG signals: (**a**) *A_cf_i_*/*A_cf__*_1_ and (**b**) $\bar{\phi }$_0*i*_ − $\bar{\phi }$_01_.

Given that pulse amplitude is highly sensitive to contact pressure, the normalized amplitude and initial phase difference of each harmonic—relative to the first harmonic—were used to compare the two groups. As shown in Figure 10a, the normalized amplitude of the second harmonic is consistently larger than that of the third harmonic, irrespective of AF or non-AF status. No significant difference in the normalized amplitudes of the two harmonics was observed between the AF and non-AF groups, and the amplitudes varied considerably across subjects within each group.

Figure 10b shows the initial phase differences of the two harmonics, which also do not differ between AF and non-AF subjects. For the non-AF group, the initial phase of the second harmonic remains larger than that of the third harmonic. In contrast, for the AF group, the initial phase of the third harmonic exceeds that of the second harmonic in some subjects ($\bar{\phi }$_03_ > $\bar{\phi }$_02_ for Subjects 2, 4, 8, 11, 12, 14, 15). While the initial phase differences in the non-AF group exhibit only slight variation between subjects, they fluctuate substantially among the AF subjects. As shown in Figure 5e and Figure 9b, time-varying HR contributes to preserving the dicrotic notch in the diastolic portion in Subjects 2 and 4, as compared to the APWs of Subjects 3 and 5. To further validate whether time-varying HR contributes to this effect on the dicrotic notch for other PPG signals with $\bar{\phi }$_03_ > $\bar{\phi }$_02_, the APWs with time-varying HR and constant HR for Subjects 11, 14, and 15 were plotted (see Figure A2 in Appendix A). It becomes clear that only when $\bar{\phi }$_03_ significantly exceeds $\bar{\phi }$_02_ does time-varying HR contribute to preserving the dicrotic notch in the diastolic portion.

**Figure 11:**
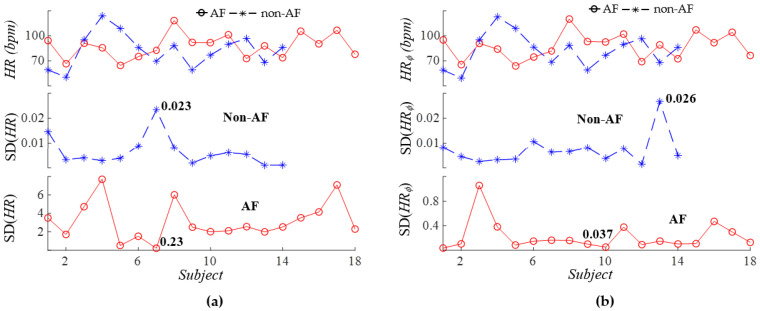
Comparison of AF versus non−AF average HR and standard deviation extracted from the three harmonics: (**a**) *HR* and *SD* (*HR*) and (**b**) *HR_ϕ_* and *SD* (*HR_ϕ_*).

Figure 11a compares the HR and standard deviation of HR extracted from *f_i_*(*t*) (averaged from the three harmonics, see Equation (16) and Figure 3) between the two groups. Figure 11b compares the HR and standard deviation of HRV extracted from *ϕ*_0*i*_(*t*) (averaged from the three harmonics, see Equation (17) and Figure 3) between the two groups. Both *HR* and *HR_ϕ_* do not differ between the two groups. Yet, *SD* (*HR*) in the AF group (lowest value: 0.23) is well above that in the non-AF group (highest value: 0.023). *SD* (*HR_ϕ_*) in the AF group is also large in the AF group (lowest value: 0.037), as compared to the non-AF group (highest value: 0.027).

**Figure 12:**
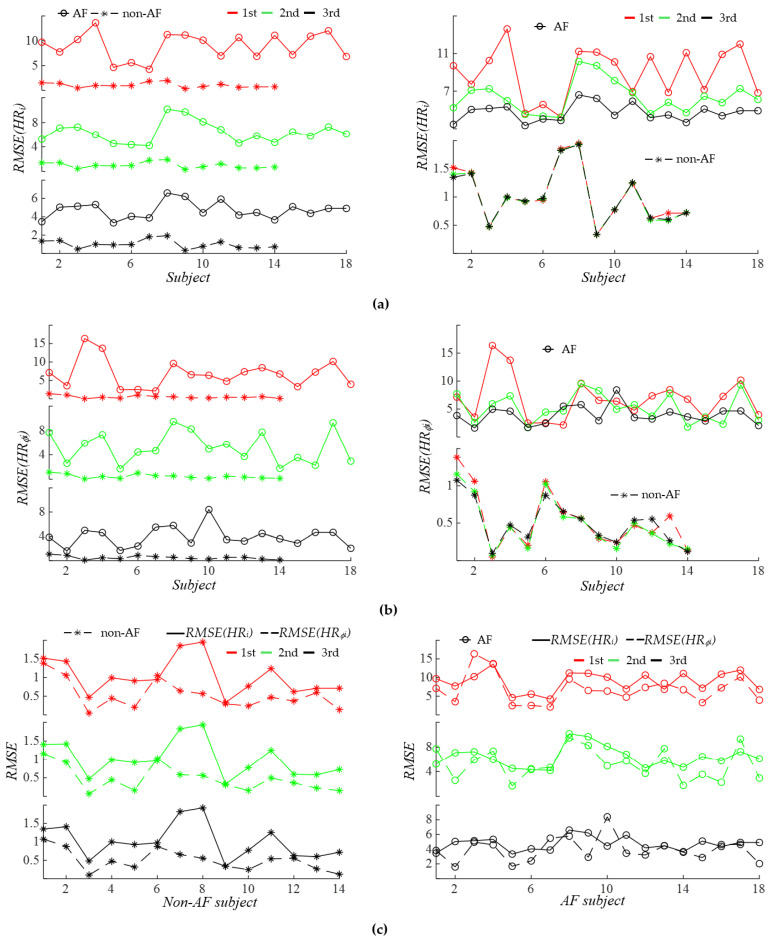
Comparison of AF versus non−AF HRV: (**a**) total HRV, RMSE (*HR_i_*); (**b**) HRV accounting solely for respiration RMSE (*HR_ϕi_*); and (**c**) comparison of *RMSE* (*HR_i_*) with *RMSE* (*HR_ϕi_*) in the non-AF group and the AF group.

Figure 12 compares the HRV extracted from *f_i_*(*t*) and *ϕ*_0*i*_(*t*) between the two groups. As shown in Figure 12a, *RMSE* (*HR_i_*) represents the toal HRV, accounting for both respiration and PF. Regardless of the harmonics, *RMSE* (*HR_i_*) is always larger in the AF group as compared to the non-AF group. While *RMSE* (*HR_i_*) remains nearly identical for all the harmonics in the non-AF group, it swings dramatically between the harmonics in the AF group. As shown in Figure 12b, *RMSE* (*HR_ϕi_*) represents the HRV accounting solely for respiration. Regardless of the harmonics, *RMSE* (*HR_ϕi_*) is always larger in the AF group as compared to the non-AF group. While *RMSE* (*HR_ϕi_*) remain very similar between the harmonics in the non-AF group, it swings greatly between the harmonics and does not display any changing trend with harmonic order.

Figure 12c compares *RMSE* (*HR_i_*) with *RMSE* (*HR_ϕi_*) in each group. From the physiological perspective, *RMSE* (*HR_i_*) should always be higher than *RMSE* (*HR_ϕi_*), in that the former accounts for both respiration and PF, whereas the latter accounts for only respiration. For some subjects in the AF group, *RMSE* (*HR_ϕi_*) is noticeably higher than *RMSE* (*HR_i_*), which can attribute to the relatively large effect of MA on *f_i_*(*t*). Theoretically, the difference between *RMSE* (*HR_i_*) and *RMSE* (*HR_ϕi_*) should represent the effect of PF on the HRV. However, given that (1) *RMSE* (*HR_i_*) is affected by MA and (2) this difference shown in Figure 12c is not substantial for all the subjects, the effect of MA on *RMSE* (*HR_i_*) cannot be neglected, rendering this difference unreliable. Consequently, it remains unclear which factor—respiration or PF—makes more of a contribution to HRV.

**Figure 13:**
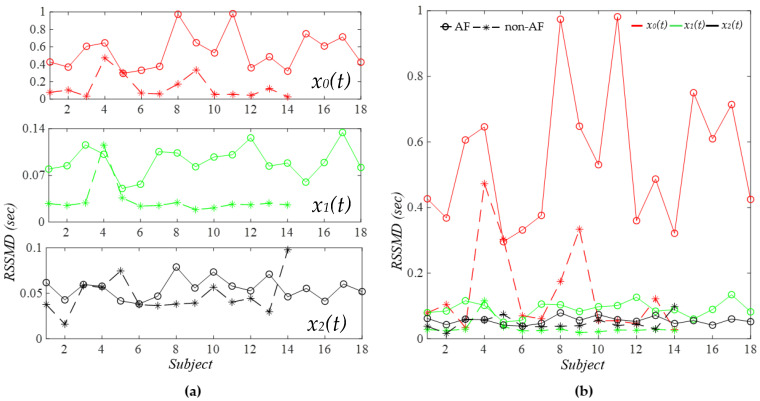
Comparison of AF versus non−AF RSSMD with no MA removal: (**a**) separate plots for *x*_0_(*t*), *x*_1_(*t*), and *x*_2_(*t*) and (**b**) one plot for *x*_0_(*t*), *x*_1_(*t*), and *x*_2_(*t*).

**Figure A1:**
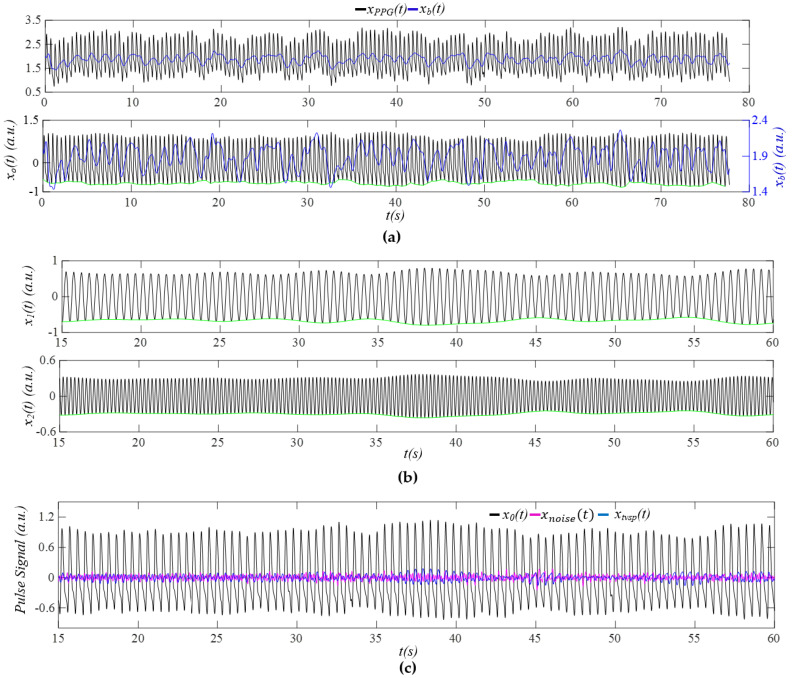
The PPG signal (segment: 730–810 s) of Subject 4 in the non−AF group: (**a**) *x_PPG_*(*t*), *x*_0_(*t*), and *x_b_*(*t*) (green line: baseline of *x*_0_(*t*)); (**b**) *x*_1_(*t*) and *x*_2_(*t*) containing *x_tvsp_*(*t*) (green line: baseline of *x*_1_(*t*) and *x*_2_(*t*)); (**c**) *x*_0_(*t*), *x_tvsp_*(*t*), and *x_noise_*(*t*).

Figure 13 compared the HRV calculated in the time domain, in terms of RMSSD (Root Mean Square of Successive Differences), between the two groups based on the results in Figure 7 and Figure 8. As shown in Figure 13a, the calculated RMSSD from *x*_0_(*t*) and *x*_1_(*t*) is overall larger in the AF group as compared to the non-AF group. In particular, if the RMSSD from *x*_1_(*t*) is used for AF detection, only Subject 4 is a false positive. As shown in Figure A1 (see Appendix A), the reason for this subject being falsely positive is simply due to large MA in the PPG signal. As with RMSSD from *x*_2_(*t*), as pointed out earlier, its small amplitude makes it extremely sensitive to MA. As shown in Figure 13b, RMSSD from *x*_0_(*t*) is overall much larger than its counterparts from *x*_1_(*t*) and *x*_2_(*t*), which might stem from *x_noise_*(*t*) in *x*_0_(*t*).

**Figure 14:**
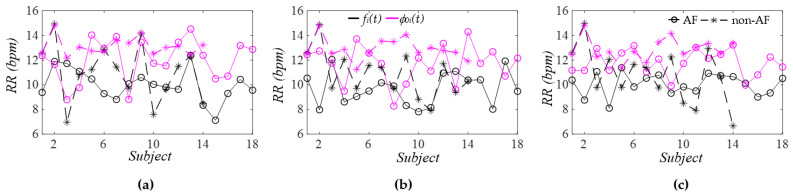
Comparison of AF versus non−AF RR extracted from *f_i_*(*t*) and *ϕ*_0*i*_(*t*): (**a**) the first harmonic; (**b**) the second harmonic; and (**c**) the third harmonic.

Figure 14 compares the respiration rate (RR) extracted from *f_i_*(*t*) and *ϕ_i_*(*t*) of the three harmonics. Regardless of the harmonic or AF/non-AF status, the RR derived from *ϕ_i_*(*t*) is consistently higher than that obtained from *f_i_*(*t*). Since *ϕ_i_*(*t*) is more robust to MA than *f_i_*(*t*), respiration modulation (RM) is extracted exclusively from *ϕ_i_*(*t*). Notably, the extracted RR does not differ between harmonics for either the AF or the non-AF group. There is also no difference in RR between the two groups.

**Figure 15:**
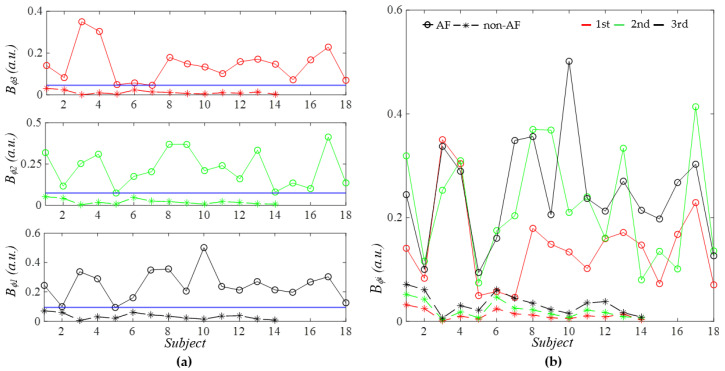
Comparison of AF versus non-AF RM: mean (*B_ϕi_*) extracted from *ϕ*_0*i*_(*t*). (**a**) Separate plots for each harmonic and (**b**) one plot for all the three harmonics.

As shown in Figure 15, the respiration modulation (RM) of each harmonic is lower in the non-AF group compared to the AF group. Furthermore, RM remains relatively consistent across subjects in the non-AF group, whereas it varies substantially between subjects in the AF group. Interestingly, while RM in the non-AF group overall exhibits an increasing trend with harmonic order, such a trend is diminished in the AF group. Note that Subject 5 in the AF group exhibits the lowest RM, which is consistent with the other corresponding results: the lowest *RMSE* (*HR_i_*) and *RMSE* (*HR_ϕi_*) in Figure 12 and the small difference between *x_tf_*(*t*) and *x_cf_*(*t*) in Figure 9.

## 4. Discussion

The comparison of this method with related time–frequency approaches, as well as with machine learning and deep learning techniques, has been presented in [25] and is not repeated here. Briefly, compared with the other time–frequency approaches in the literature, the SDOF-TF method [25] is built upon the SDOF model of MA, which leads to the following findings regarding the effects of TVSP on instant parameters: (1) TVSP greatly swings the instant amplitude of each harmonic; (2) TVSP slightly affects the instant frequency of each harmonic; (3) TVSP has almost no effect on the instant initial phase of each harmonic; (4) the instant frequency of each harmonic carries both the respiration signal and PF; and (5) the instant initial phase of each harmonic carries only the respiration signal. Based on these findings, the SDOF-TF method was developed to extract the corresponding parameters for a pulse signal. Among all the studies that have evaluated signal-processing methods for AF detection using the same dataset, the proposed SDOF-TF method is, to the best of the authors’ knowledge, the first to achieve perfect AF detection accuracy. This section thus concentrates on the implications of the analyzed results and the limitations in the present study.

### 4.1. Implications of the Analyzed Results

#### 4.1.1. Entangled MA, Noise, and HRV

The analysis in Section 3 reveals entangled relationships among MA, HRV, and noise. As shown in Figure 2, physiological condition appears both in MA and in the true pulse signal. In the true pulse signal, it manifests solely as HRV (i.e., time-varying frequency), whereas in MA (i.e., *x_tvsp_*(*t*)), it is complicated by the contributions of sensor fixture and the TCS stack. Nevertheless, as demonstrated in Figure 6 and Figure A1, the temporal variation in *x_tvsp_*(*t*) is dominated by physiological condition. Similarly, the noise in a PPG signal *x_noise_*(*t*) is correlated with the PPG signal itself and *x_tvsp_*(*t*). For the non-AF subject, the temporal variation in *x_noise_*(*t*) follows that of *x_tvsp_*(*t*), whereas for the AF subject, both *x_noise_*(*t*) and *x_tvsp_*(*t*) exhibit random time-varying behavior.

#### 4.1.2. APW with Time-Varying HR Versus APW with Constant HR

**Figure A2:**
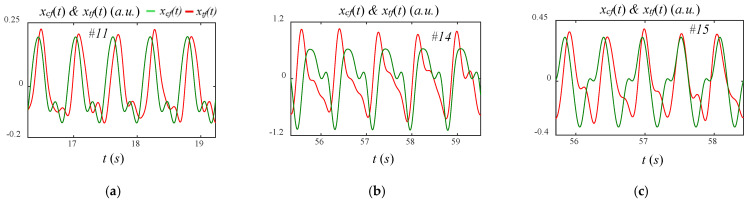
Comparison of APW between time-varying HR and constant HR: (**a**) Subject 11; (**b**) Subject 14; (**c**) Subject 15 in the AF group.

As shown in Figure 4e, Figure 5e and Figure 9, and A2, large time-varying HR causes significant variation in APW between pulse cycles in the AF subjects, whereas small time-varying HR in the non-AF subjects translates to slight variation in APW between pulse cycles. To reveal the role of time-varying HR in APW, we reconstructed the APW with constant HR for both groups. As shown in Figure 5e and Figure 9b, and A2, large time-varying HR can greatly affect APW, as compared to its counterpart with constant HR.

Since the visual examination of the APW between AF and non-AF subjects does not reveal any distinguishing features, we further compare their normalized amplitudes and initial phases of the second and third harmonics, relative to the first harmonic. As illustrated in Figure 10, neither the amplitudes nor the initial phases of the harmonics effectively separate AF from non-AF subjects. Notably, in Subjects 2 and 4 of the AF group, the initial phase of the third harmonic exceeds that of the second harmonic (i.e., $\bar{\phi }$_03_ > $\bar{\phi }$_02_), which may explain the appearance of a notch in the systolic portion of the pulse cycle in the APW with constant HR. To further verify whether $\bar{\phi }$_03_ > $\bar{\phi }$_02_ underlies the appearance of the dicrotic notch in the systolic portion, Subjects 11, 14, and 15 with $\bar{\phi }$_03_ > $\bar{\phi }$_02_ in Figure 10b were analyzed. Their APWs with time-varying HR and constant HR show that only when $\bar{\phi }$_03_ significantly exceeds $\bar{\phi }$_02_ does time-varying HR contribute to preserving the dicrotic notch in the diastolic portion. These results suggest that time-varying HR contributes to maintaining the pulse waveform near its normal shape when the initial phase of the third harmonic is substantially higher than that of the second harmonic in an AF subject.

#### 4.1.3. HR and HRV Derived from Instant Frequency and Instant Initial Phase

As shown in Equation (9), the instant frequency *f_i_*(*t*) of a harmonic accounts for both respiration and PF. Accordingly, the HR derived from *f_i_*(*t*) represents the total HR. In contrast, the HR derived from the instant initial phase *ϕ_i_*(*t*) excludes the contribution of PF to the HR. Yet, based on the SDOF model of MA [25], MA has almost no effect on *ϕ_i_*(*t*) but does influence *f_i_*(*t*) to some extent.

As shown in Figure 11a, the average HR *HR* derived from *f_i_*(*t*) of the three harmonics does not exhibit any clear difference between AF and non-AF subjects. In contrast, the standard deviation of HR *SD* (*HR*) across the harmonics effectively separates AF from non-AF subjects. As illustrated in Figure 4 and Figure 5, the effect of MA on *f_i_*(*t*) is similar across the harmonics. As such, the large *SD* (*HR*) observed in the AF group is believed to reflect the elevated HRV characteristic of AF. As shown in Figure 11b, the average HR *HR_ϕ_* derived from *ϕ_i_*(*t*) shows no noticeable difference between the two groups. However, the standard deviation of HR *SD* (*HR_ϕ_*) provides clear separation between AF and non-AF subjects. This large *SD* (*HR_ϕ_*) in the AF group is believed to arise from substantial variation in respiration modulation (RM) across the harmonics, as evidenced in Figure 15.

As shown in Figure 13, the HRV *RSSMD* extracted in the time domain from *x*_0_(*t*) fails to effectively distinguish AF from non-AF subjects, highlighting the necessity of removing MA and noise. Even in the absence of noise, the HRV extracted from *x*_1_(*t*) misdiagnosed one non-AF subject due to large MA in the signal. These findings stress that the effective removal of MA and noise is critical for accurate AF detection using time-domain HRV.

#### 4.1.4. Identified Indices for AF Detection and Observed Physiological Implications

In addition to *SD* (*HR*) and *SD* (*HR_ϕ_*), as shown in Figure 12, both the total HRV *RMSE* (*HR_i_*) derived from *f_i_*(*t*) and the HRV *RMSE* (*HR_ϕi_*) derived from *ϕ_i_*(*t*) demonstrate clear separation between AF and AF subjects for each harmonic. Furthermore, as illustrated in Figure 15, RM mean (*B_ϕi_*(*t*)) derived from *ϕ_i_*(*t*) also effectively distinguishes AF from non-AF subjects.

In summary, three categories of parameters extracted from an at-rest PPG signal can be used for AF detection:

(1) *SD* (*HR*) and *SD* (*HR_ϕ_*)*;*
(2) *RMSE* (*HR_i_*) and *RMSE* (*HR_ϕi_*) of each harmonic;
(3) *mean* (*B_ϕi_*(*t*)) of each harmonic.

The mean, standard deviation, median, and range of these indices are summarized in Table A4 in Appendix A, showing non-overlapping values between the AF and non-AF groups, whereas the other extracted parameters exhibit overlapping values between the two groups. These parameters serve as effective indices for AF detection, as each captures aspects of HRV within a PPG signal that is largely free of MA and noise.

Furthermore, three physiological implications emerge from this study:

(1) AF increases RM for each harmonic;
(2) AF disrupts the increasing trend of RM with harmonic order;
(3) Elevated HRV contributes to maintaining the pulse waveform near its normal shape, when AF causes the initial phase of the third harmonic to significantly exceed that of the second harmonic.

Finally, it is worth noting that the increase in RM in AF does not necessarily indicate that respiration plays a more dominant role in determining HRV compared with PF.

### 4.2. Study Limitations

This study has six limitations. First, the complexity of optical transduction in a PPG sensor is substantially simplified. As demonstrated by the analyzed results, this simplification does not obscure the expected differences in HRV between AF and non-AF subjects. Second, the SDOF-TF method is applicable only to at-rest PPG signals and is unsuitable for wearable PPG signals (i.e., PPG signals recorded during activities). As described in [25], the frequency of MA during activities falls into the frequency range of the pulse signal itself and may vary between pulse cycles. Based on the SDOF model of MA, as seen in Equation (3), such MA will generate multiple signals at different frequencies and thus contaminate those harmonics whose frequencies are close to theirs [25]. Furthermore, activities can easily cause intermittent tissue–sensor contact, create nonlinearity in the TCS stack (manifested as abrupt changes in the pulse waveform), and severely distort pulse cycles.

Third, the nonlinearity of the TCS stack is neglected in the analysis. As long as there is no abrupt change in the pulse waveform of a PPG signal, tissue–sensor contact remains consistent, and the assumption of linearity in the TCS stack is practical. As noted in Section 2.2, segments of the PPG signals were selected to avoid abrupt changes. The results presented here indicate that this assumption does not affect the identification of indices for AF detection. It is worth noting that the analysis of PPG signals with abrupt changes may increase the risk of false-positive detections. False-negative detections are unlikely unless the PPG sensor malfunctions.

Fourth, no comparison of the SDOF-TF method with other time–frequency methods reported in the literature was conducted. Since the SDOF-TF method achieves 100% detection accuracy, such a comparison was deemed unnecessary. Fifth, the sample size of the dataset is small. In future work, the identified indices for AF detection and the observed physiological implications will need to be further validated using datasets with larger sample sizes.

Lastly, the effect of the system parameters *m*_0_, *k*_0_, and *c*_0_ of the TCS stack on a PPG signal is not explored. Based on its working principle [25], the SDOF-TF method is applicable to various sensor types: tactile sensors, accelerometers, PPG sensors, and even an ultrasound probe for measuring radial arterial wall displacement [31]. As illustrated in Figure 2, the nominal values of *m*_0_, *k*_0_, and *c*_0_ represent the collective behavior of the sensor, contact pressure, and the individual (i.e., tissue). Clearly, TVSP—*m*(*t*), *k*(*t*), and *c*(*t*)—of the TCS stack is related not only to baseline drift (BD) but also to *m*_0_, *k*_0_, and *c*_0_. At the same time, BD is also related to *m*_0_, *k*_0_, and *c*_0_ as well as the individual (i.e., physiological condition). All these system parameters of the TCS stack vary with sensor type, individual, and contact pressure. For instance, high contact pressure translates to low BD and, consequently, low TVSP. Th effect of contact pressure on a measured pulse signal is multifaceted, and a detailed discussion can be found in the literature [25,26,27,28,31]; it is beyond the scope of this study. Given that there are six unknowns but only one measured pulse signal, there is currently no method for identifying their exact values. However, as demonstrated in this study, not knowing the values of these parameters does not affect the removal of the MA and noise of a PPG signal for AF detection.

## 5. Conclusions

In this study, the SDOF-TF method was applied to at-rest PPG signals from the MIMIC PERform AF dataset to evaluate its effectiveness in removing MA and noise and detecting AF. The arterial pulse waveform (APW)—characterized by normalized amplitudes and relative initial phase differences with respect to the first harmonic—was found to be ineffective in distinguishing AF from non-AF subjects. In contrast, three categories of time–frequency parameters enabled AF detection with 100% accuracy: (1) respiration modulation (RM), (2) total HRV derived from instant frequency and HRV attributable solely to respiration derived from instant initial phase, and (3) the standard deviation of total HR and HR accounting solely for respiration across harmonics.

Compared with non-AF subjects, RM was increased in AF subjects. RM exhibited an increasing trend with harmonic order in the non-AF group, whereas this trend was diminished in the AF group. Elevated HRV was also found to contribute to maintaining the pulse waveform near its normal shape (i.e., the dicrotic notch in the diastolic portion) in those AF subjects where the initial phase of the third harmonic substantially exceeds that of the second harmonic.

## Data Availability

The data in the MIMIC PERform AF Dataset [21] are used.

## Abbreviations

The following abbreviations are used in this manuscript:

AF	Atrial fibrillation
APW	Arterial pulse waveform
BD	Baseline drift
SDOF	Single-degree-of-freedom
TVSPs	Time-varying system parameters
MA	Motion artifacts
SDOF-TF	Single-degree-of-freedom time–frequency
HVD	Hilbert vibration transform
PFs	Other physiological factors
FFT	Fast Fourier transform

## Appendix A

**Table A1:** Selected segments: *t_start_* − *t_end_* of the PPG signals in (**a**) the AF group and (**b**) the non-AF group used in the analysis.

(a) AF Subjects																			
**Subject**	**1**	**2**	**3**	**4**	**5**	**6**	**7**	**8**	**9**	**10**	**11**	**12**	**13**	**14**	**15**	**16**	**17**	**18**	**19**
*t_start_* (s)	70	955	5	940	37	-	180	198	100	30	185	20	16	5	1007	510	320	470	180
*t_end_* (s)	150	1035	85	1020	117	-	260	278	180	110	265	100	96	85	1087	590	400	550	260
**(b) Non-AF subjects**																			
**Subject**	**1**	**2**	**3**	**4**	**5**	**6**	**7**	**8**	**9**	**10**	**11**	**12**	**13**	**14**	**15**	**16**			
*t_start_* (s)	450	945	395	730	180	1105	132	64	990	595	1070	-	780	-	790	740			
*t_end_* (s)	530	1025	475	810	260	1185	212	144	1080	675	1150	-	860	-	870	820			

**Table A2:** The numerical values of the extracted parameters for the non-AF group.

Subject	1	2	3	4	5	6	7	8	9	10	11	12	13	14
A_cf2_/A_cf1_ (a.u.)	0.3928	0.5285	0.3820	0.4501	0.4075	0.3591	0.6803	0.6078	0.4994	0.4484	0.5643	0.5284	0.5501	0.4040
A_cf3_/A_cf1_ (a.u.)	0.2415	0.2779	0.0861	0.1357	0.1094	0.0645	0.3936	0.2553	0.2606	0.1328	0.1843	0.1572	0.1722	0.0955
$\bar{\phi }$_02_ − $\bar{\phi }$_01_ (*rad*)	4.972	4.929	4.862	4.896	5.347	4.572	5.098	5.078	4.880	4.853	4.851	4.848	4.811	4.777
$\bar{\phi }$_03_ − $\bar{\phi }$_01_ (*rad*)	4.156	3.325	4.405	4.215	4.175	3.135	3.511	4.072	3.454	3.980	3.871	3.599	3.566	3.346
*HR* (*bpm*)	59.11	50.42	95.12	124.00	108.66	85.97	69.44	88.34	59.17	76.73	89.89	96.75	68.27	86.11
*SD* (*HR*)	0.0146	0.0033	0.0040	0.0029	0.0038	0.0088	0.0235	0.0082	0.0020	0.0048	0.0061	0.0054	0.0010	0.0011
*HR_ϕ_* (*bpm*)	58.90	49.27	95.15	122.75	108.90	86.44	68.33	88.64	59.02	76.59	89.71	96.47	68.02	86.23
*SD* (*HR_ϕ_*)	0.0082	0.0045	0.0025	0.0032	0.0035	0.0107	0.0063	0.0066	0.0081	0.0038	0.0078	0.0014	0.0267	0.0049
RMSE (*HR*_1_)	1.519	1.435	0.471	0.992	0.912	0.944	1.851	1.951	0.333	0.770	1.240	0.621	0.714	0.716
RMSE (*HR*_2_)	1.405	1.421	0.473	0.986	0.916	0.964	1.827	1.934	0.333	0.775	1.246	0.594	0.584	0.724
RMSE (*HR*_3_)	1.349	1.413	0.480	1.002	0.927	0.974	1.823	1.927	0.336	0.773	1.254	0.627	0.600	0.719
*RMSE* (*HR*_f1_)	1.384	1.062	0.050	0.447	0.206	1.055	0.645	0.567	0.296	0.242	0.471	0.373	0.597	0.140
*RMSE* (*HR*_f2_)	1.157	0.923	0.071	0.446	0.167	1.024	0.584	0.562	0.313	0.161	0.496	0.364	0.224	0.155
*RMSE* (*HR*_f3_)	1.075	0.875	0.099	0.473	0.317	0.875	0.656	0.556	0.337	0.247	0.539	0.554	0.266	0.121
*RMSSD* (*x*_0_(*t*)) (*sec*)	0.0788	0.1041	0.0340	0.4728	0.3034	0.0699	0.0607	0.1741	0.3338	0.0553	0.0546	0.0453	0.1216	0.0274
*RMSSD* (*x*_1_(*t*)) (*sec*)	0.0276	0.0250	0.0287	0.1152	0.0363	0.0238	0.0249	0.0292	0.0186	0.0212	0.0265	0.0257	0.0283	0.0257
*RMSSD* (*x*_2_(*t*)) (*sec*)	0.0376	0.0160	0.0584	0.0564	0.0746	0.0375	0.0363	0.0381	0.0392	0.0567	0.0402	0.0445	0.0295	0.0978
*RR*(*f*_1_(*t*)) (*bpm*)	12.564	14.936	6.963	10.818	11.229	12.876	11.447	9.731	14.130	7.591	9.621	11.505	12.325	8.485
*RR*(*f*_2_(*t*)) (*bpm*)	12.586	14.875	9.737	12.062	9.711	11.578	11.393	9.659	12.322	8.809	7.909	11.705	9.385	10.279
*RR*(*f*_3_(*t*)) (*bpm*)	12.580	14.983	9.778	12.044	9.784	11.652	11.377	9.739	12.259	8.502	7.910	12.940	10.619	6.664
*RR*(*ϕ*_01_(*t*)) (*bpm*)	12.579	14.791	12.154	13.063	12.748	12.662	13.623	13.386	14.221	12.503	13.019	13.130	12.451	13.229
*RR*(*ϕ*_02_(*t*)) (*bpm*)	12.542	14.792	12.523	12.861	11.242	12.614	13.543	13.495	14.077	12.588	13.005	12.770	12.630	11.920
*RR*(*ϕ*_03_(*t*)) (*bpm*)	12.470	14.797	12.272	12.654	11.395	12.687	11.812	13.450	14.162	12.488	13.041	13.352	12.558	13.369
*B*_*ϕ*1_ (a.u.)	0.0317	0.0247	0.0011	0.0099	0.0045	0.0239	0.0144	0.0120	0.0068	0.0053	0.0106	0.0087	0.0138	0.0032
*B*_*ϕ*2_ (a.u.)	0.0518	0.0427	0.0031	0.0180	0.0068	0.0466	0.0252	0.0221	0.0142	0.0074	0.0219	0.0170	0.0088	0.0070
*B*_ϕ3_ (a.u.)	0.0712	0.0610	0.0064	0.0301	0.0215	0.0608	0.0444	0.0348	0.0226	0.0155	0.0356	0.0379	0.0168	0.0081

**Table A3:** The numerical values of the extracted parameters from the AF group dataset.

Subject	1	2	3	4	5	6	7	8	9	10	11	12	13	14	15	16	17	18
A_cf2_/A_cf1_ (a.u.)	0.3447	0.3903	0.4647	0.7498	0.3704	0.4152	0.2976	0.2935	0.3408	0.3374	0.5247	0.6052	0.4193	0.5742	0.4380	0.5186	0.3448	0.5004
A_cf3_/A_cf1_ (a.u.)	0.1225	0.2027	0.1635	0.3104	0.1212	0.1425	0.1062	0.0841	0.1382	0.0969	0.1395	0.2956	0.1616	0.2378	0.1108	0.1595	0.1144	0.3475
$\bar{\phi }$_02_ − $\bar{\phi }$_01_ (*rad*)	4.940	2.245	4.934	0.462	4.550	4.810	5.114	5.031	4.132	6.032	4.300	4.888	5.308	5.575	1.138	4.711	4.675	3.929
$\bar{\phi }$_03_ − $\bar{\phi }$_01_ (*rad*)	1.508	5.788	4.311	2.688	3.074	3.908	3.450	5.283	0.518	3.653	5.570	5.045	3.803	6.114	5.566	3.147	2.292	0.805
*HR* (*bpm*)	94.34	66.51	91.15	85.70	64.51	75.14	82.65	118.35	92.18	91.84	101.28	72.75	87.99	73.77	105.53	90.45	106.65	77.93
*SD* (*HR*)	3.513	1.725	4.728	7.706	0.528	1.527	0.231	6.017	2.506	2.023	2.122	2.566	2.000	2.534	3.530	4.158	7.090	2.307
*HR*_*ϕ*_ (*bpm*)	95.08	65.43	90.92	84.11	63.94	74.60	81.79	120.17	93.13	92.65	102.09	69.00	89.13	72.50	107.11	91.82	104.31	76.44
*SD* (*HR_ϕ_*)	0.0373	0.1053	1.0546	0.3826	0.0861	0.1494	0.1642	0.1600	0.1016	0.0528	0.3774	0.0933	0.1506	0.1035	0.1085	0.4708	0.2989	0.1301
RMSE (*HR*_1_)	9.702	7.735	10.256	13.616	4.631	5.585	4.245	11.233	11.148	10.107	6.948	10.666	6.864	11.092	7.165	10.912	12.010	6.824
RMSE (*HR*_2_)	5.242	7.105	7.258	5.982	4.529	4.344	4.203	10.162	9.710	8.122	6.840	4.602	5.813	4.714	6.463	5.780	7.274	6.122
RMSE (*HR*_3_)	3.470	5.052	5.161	5.339	3.345	4.051	3.885	6.607	6.227	4.438	5.935	4.185	4.472	3.670	5.110	4.380	4.926	4.922
*RMSE* (*HR*_*f*1_)	7.131	3.552	16.365	13.729	2.487	2.541	2.145	9.596	6.549	6.400	4.770	7.355	8.446	6.745	3.306	7.271	10.156	3.964
*RMSE* (*HR*_*f*2_)	7.723	2.602	5.926	7.354	1.708	4.446	4.663	9.511	8.290	4.954	5.758	3.722	7.774	1.781	3.557	2.288	9.309	2.965
*RMSE* (*HR*_*f*3_)	3.835	1.598	4.969	4.609	1.686	2.408	5.504	5.781	2.914	8.413	3.461	3.224	4.484	3.597	2.870	4.640	4.654	2.038
*RMSSD* (*x*_0_(*t*)) (*sec*)	0.4265	0.3680	0.6061	0.6460	0.2958	0.3315	0.3761	0.9740	0.6476	0.5305	0.9817	0.3598	0.4867	0.3214	0.7500	0.6096	0.7140	0.4247
*RMSSD* (*x*_1_(*t*)) (*sec*)	0.0794	0.0845	0.1156	0.1017	0.0503	0.0567	0.1055	0.1036	0.0829	0.0975	0.1009	0.1262	0.0839	0.0884	0.0599	0.0893	0.1343	0.0819
*RMSSD* (*x*_2_(*t*)) (*sec*)	0.0618	0.0427	0.0596	0.0578	0.0416	0.0383	0.0468	0.0789	0.0558	0.0734	0.0579	0.0527	0.0710	0.0461	0.0553	0.0412	0.0602	0.0518
*RR*(*f*_1_(*t*)) (*bpm*)	9.381	11.893	11.725	11.084	10.466	9.278	8.809	10.074	10.593	10.034	9.796	9.629	12.378	8.347	7.122	9.294	10.428	9.563
*RR*(*f*_2_(*t*)) (*bpm*)	10.529	7.975	12.044	8.612	9.051	9.494	10.166	9.893	8.314	7.812	8.147	10.950	11.095	10.381	10.400	8.021	11.919	9.472
*RR*(*f*_3_(*t*)) (*bpm*)	10.377	8.762	11.065	8.105	11.381	9.829	10.534	10.788	9.316	9.829	9.499	10.934	10.737	10.657	10.127	9.010	9.335	10.516
RR(*ϕ*_01_(*t*)) (*bpm*)	12.365	11.647	8.791	9.762	14.032	12.963	13.909	8.806	13.459	11.748	11.538	13.460	14.516	12.389	10.486	10.704	13.186	12.870
RR(*ϕ*_02_(*t*)) (*bpm*)	12.451	12.739	11.758	9.493	13.714	12.568	11.714	8.276	10.055	12.178	11.119	13.351	9.617	14.296	11.741	12.675	10.706	12.154
RR(*ϕ*_03_(*t*)) (*bpm*)	11.180	11.160	12.944	11.176	12.577	13.171	11.060	12.079	9.919	11.731	13.020	12.143	12.456	13.229	9.974	10.805	12.245	11.447
*B*_*ϕ*1_ (a.u.)	0.1409	0.0830	0.3503	0.3040	0.0496	0.0575	0.0462	0.1792	0.1487	0.1337	0.1018	0.1594	0.1713	0.1471	0.0726	0.1678	0.2290	0.0704
*B*_*ϕ*2_ (a.u.)	0.3191	0.1161	0.2529	0.3098	0.0741	0.1750	0.2036	0.3699	0.3689	0.2100	0.2401	0.1604	0.3339	0.0800	0.1350	0.1011	0.4138	0.1358
*B*_*ϕ*3_ (a.u.)	0.2443	0.1002	0.3375	0.2894	0.0941	0.1603	0.3487	0.3563	0.2057	0.5015	0.2369	0.2126	0.2702	0.2141	0.1975	0.2676	0.3029	0.1263

**Table A4:** The mean, median, standard deviation (SD), and range of all the extracted parameters from the PPG signals, with the identified indices for AF detection highlighted in bold.

	AF Group				Non-AF Group			
Extracted Parameters	Mean	Median	SD	Range	Mean	Median	SD	Range
*A*_*cf*2_/*A*_*cf*1_ (a.u.)	0.441	0.417	0.121	0.293–0.750	0.486	0.475	0.095	0.359–0.680
*A*_*cf*3_/*A*_*cf*1_ (a.u.)	0.170	0.141	0.078	0.084–0.348	0.183	0.165	0.092	0.064–0.394
$\bar{\phi }$_02_ − $\bar{\phi }$_01_ (*rad*)	4.265	4.761	1.490	0.462–6.032	4.912	4.871	0.179	4.572–5.347
$\bar{\phi }$_03_ − $\bar{\phi }$_01_ (*rad*)	3.696	3.728	1.698	0.518–6.114	3.772	3.735	0.400	3.135–4.405
*HR* (*bpm*)	87.707	89.217	14.529	64.51–118.35	82.713	86.040	20.398	50.42–124.00
***SD*** **(*HR*)**	3.156	2.520	2.084	0.231–7.706	0.006	0.004	0.006	0.001–0.024
*HR_ϕ_* (*bpm*)	87.456	90.026	15.412	63.94–120.17	82.458	86.330	20.473	49.27–122.75
***SD*** **(*HR_ϕ_*)**	0.224	0.140	0.241	0.037–1.055	0.007	0.006	0.006	0.001–0.027
**RMSE (*HR*_1_)**	8.930	9.905	2.720	4.245–13.616	1.033	0.928	0.498	0.333–1.951
**RMSE (*HR*_2_)**	6.348	6.052	1.735	4.203–10.162	1.013	0.940	0.492	0.333–1.934
**RMSE (*HR*_3_)**	4.732	4.697	0.923	3.345–6.607	1.015	0.951	0.483	0.336–1.927
***RMSE*** **(*HR_f_*_1_)**	6.806	6.647	3.877	2.145–16.365	0.538	0.459	0.389	0.050–1.384
***RMSE*** **(*HR_f_*_2_)**	5.241	4.809	2.582	1.708–9.511	0.475	0.405	0.345	0.071–1.157
***RMSE*** **(*HR_f_*_3_)**	3.927	3.716	1.685	1.598–8.413	0.499	0.506	0.295	0.099–1.075
*RMSSD* (*x*_0_(*t*)) (*sec*)	0.547	0.509	0.211	0.296–0.982	0.138	0.074	0.136	0.027–0.473
*RMSSD* (*x*_1_(*t*)) (*sec*)	0.091	0.089	0.022	0.050–0.134	0.033	0.026	0.024	0.019–0.115
*RMSSD* (*x*_2_(*t*)) (*sec*)	0.055	0.056	0.011	0.038–0.079	0.047	0.040	0.020	0.016–0.098
*RR*(*f*_1_(*t*)) (*bpm*)	9.994	9.915	1.291	7.122–12.378	11.016	11.338	2.342	6.963–14.936
*RR*(*f*_2_(*t*)) (*bpm*)	9.682	9.694	1.355	7.812–12.044	10.858	10.836	1.832	7.909–14.875
*RR*(*f*_3_(*t*)) (*bpm*)	10.045	10.252	0.891	8.105–11.381	10.774	10.998	2.218	6.664–14.983
RR(*ϕ*_01_(*t*)) (*bpm*)	12.035	12.377	1.742	8.791–14.516	13.111	13.041	0.721	12.154–14.791
RR(*ϕ*_02_(*t*)) (*bpm*)	11.700	11.956	1.577	8.276–14.296	12.900	12.700	0.877	11.242–14.792
RR(*ϕ*_03_(*t*)) (*bpm*)	11.795	11.905	1.025	9.919–13.229	12.893	12.670	0.893	11.395–14.797
***B_ϕ_*_1_ (a.u.)**	0.145	0.144	0.084	0.046–0.350	0.012	0.010	0.009	0.001–0.032
***B_ϕ_*_2_ (a.u.)**	0.222	0.207	0.109	0.074–0.414	0.021	0.018	0.016	0.003–0.052
***B_ϕ_*_3_ (a.u.)**	0.248	0.241	0.101	0.094–0.502	0.033	0.032	0.020	0.006–0.071
